# Erythrocytapheresis in the emergency management of severe falciparum malaria

**DOI:** 10.4103/0974-2700.62105

**Published:** 2010

**Authors:** Luciano Santana-Cabrera, Manuela Fernández Arroyo, Fayna Rodríguez González, Manuel Sánchez Palacios

**Affiliations:** Intensive Care Unit, Universitary Hospital Insular in Gran Canaria, Las Palmas de Gran Canaria, Spain

Sir,

*Plasmodium falciparum* causes over one million deaths every year. The management of severe malaria includes prompt administration of appropriate parenteral antimalarial agents and early recognition and treatment of complications. The complications include metabolic acidosis, hypoglycemia, hyperlacticacidemia, severe anemia, seizures and raised intracranial pressure, renal failure, and pulmonary edema.[1]

Red cell exchange using a cell separator (therapeutic erythrocytapheresis) has been used successfully in combination with antimalarial treatment in a large number of acute severe cases of malaria.[2–4]

We present a case report of a male patient of 38 years, an irregular immigrant of Chinese nationality who had arrived in our country in a ship that sailed from Guinea Conakry 3 weeks before the hospital admission. He was referred to our hospital from a reception center for immigrants after 1 week of evolution of fever, with deterioration of his general state, abdominal pain, vomiting, jaundice, headache, and decreased level of consciousness. He had no medical antecedents of interest. In the emergency department he had repeated convulsions and required endotracheal intubation and connection to a mechanical ventilator. His laboratory results showed thrombocytopenia, renal failure, hyponatremia, and increased liver enzymes. With the epidemiological background of the patient, malaria was suspected and a thick blood film was taken and stained for detecting *Plasmodium falciparum*. As there was a parasite infestation rate of 21%, we proceeded urgently to automated erythrocytapheresis through a dialysis catheter, aiming for replacement of 70% of his hematocrit so that the rate of parasitism would be brought down to <1%. The patient was also treated with intravenous infusions of quinine and doxycycline. The patient responded favorably. Within a few days he could be disconnected from the mechanical ventilator, and he was discharged to the ward a week later.

Isolated parasitemia above 10%, or a parasitemia above 5% associated with any additional World Health Organization - 2000 criteria of clinical severity, is indication for erythrocytapheresis.[2]

Erythrocytapheresis consists of removal of the red-cell fraction by apheresis. Plasma and leukocyte and platelet fractions are returned to the patient.[5] Current-generation automated cell-separator hardware and software allows prompt red cell exchange or erythrocytapheresis in a single continuous-flow isovolemic procedure and can achieve a dramatic reduction in parasitemia within 2 h. Erythrocytapheresis has significant advantages over exchange transfusion in terms of speed, efficiency, and maintenance of hemodynamic stability. It allows retention of plasma components such as clotting factors and may thus represent an important adjunctive therapy in severe malaria.
